# Reassessment of Childhood Peanut Allergy Reveals Tolerance

**DOI:** 10.7759/cureus.108431

**Published:** 2026-05-07

**Authors:** Shreena G Reddy, Vishaka R Hatcher, Charles K Miller

**Affiliations:** 1 Pediatrics, Joint Base Charleston, Charleston, USA; 2 Allergy and Immunology, Wilford Hall Medical Center, San Antonio, USA; 3 Allergy and Immunology, Mike O'Callaghan Military Medical Center, Nellis Air Force Base, USA

**Keywords:** allergen component testing, clinical tolerance, component-resolved diagnostics, food allergies, food allergy delabeling, oral food challenge, peanut allergy

## Abstract

Peanut allergy is commonly diagnosed in childhood and may persist as a medical label into adulthood despite the development of clinical tolerance, leading to potential misclassification and unnecessary dietary and occupational restrictions. We report a 19-year-old male patient with a childhood diagnosis of peanut allergy that precluded military enlistment in the United States despite years of reported asymptomatic peanut ingestion. Evaluation demonstrated persistent sensitization on skin prick testing, low-level peanut-specific IgE, and isolated sensitization to Ara h 8 on component-resolved diagnostics (CRD). Following structured reassessment, a supervised oral food challenge (OFC) was performed and tolerated without symptoms. The peanut allergy diagnosis was removed, and the patient was cleared for military accession. This case demonstrates that integration of clinical history, CRD, and OFC enables accurate risk stratification and safe delabeling of peanut allergy, with particular relevance in high-stakes settings.

## Introduction

Food allergy diagnoses carry significant medical, psychosocial, and occupational implications. Peanut allergy is among the most commonly reported food allergies and has historically been regarded as a lifelong condition [1]. Inaccurate or outdated food allergy labels may lead to unnecessary dietary restriction, anxiety, and healthcare burden. In the United States, these labels may also carry occupational consequences in certain settings, including military service, where a documented peanut allergy may be disqualifying under Department of Defense medical accession standards [2]. Up to 77% of Americans aged 17-24 may be ineligible for military service without a waiver, with medical conditions representing a leading cause of disqualification [3]. In high-stakes settings, optimizing diagnostic accuracy is therefore essential to distinguish sensitization from true allergy and to identify patients who may safely resume previously restricted foods.

Peanut allergy encompasses distinct clinical patterns, including primary IgE-mediated allergy associated with storage proteins such as Ara h 2 and pollen-food allergy syndrome (PFAS), which involves cross-reactive proteins such as Ara h 8 and is typically associated with milder or absent systemic clinical reactions [4].

Longitudinal studies suggest that peanut allergy is not universally permanent. In a multicenter cohort, approximately 21%-22% of individuals previously labeled peanut-allergic demonstrated tolerance on supervised oral food challenge (OFC), particularly among those with lower serologic peanut-specific IgE levels at reevaluation [4]. Importantly, sensitization on skin prick testing may persist despite clinical tolerance because allergen-specific IgE may remain detectable even in individuals who no longer develop symptoms upon exposure, highlighting the limited specificity of extract-based testing alone [4].

Component-resolved diagnostics (CRD) have emerged as an adjunctive tool to refine peanut allergy risk stratification by distinguishing sensitization to storage proteins associated with systemic reactions from cross-reactive protein components associated with a greater likelihood of clinical tolerance [5]. While CRD is increasingly incorporated into clinical practice, literature describing how these in vitro tools translate into real-world in vivo clinical decision-making and safe food allergy delabeling remains limited.

## Case presentation

A 19-year-old male patient presented to the Allergy and Immunology clinic for evaluation of a reported peanut allergy during assessment for potential military service in the United States. The reported allergy prompted referral for further evaluation. He had no history of asthma, atopic dermatitis, or other chronic medical conditions.

The patient reported that at approximately 10-11 years of age, he developed lip swelling and subjective shortness of breath within minutes of ingesting roasted peanuts. He was subsequently referred to an allergist, where peanut allergy was confirmed by skin prick testing. He was prescribed an epinephrine autoinjector and instructed to strictly avoid peanut consumption. He initially avoided peanuts but resumed ingestion around age 13 without recurrence of symptoms and continued to tolerate peanut butter regularly thereafter. He denied subsequent emergency department visits or hospitalizations related to food allergy. The patient’s longitudinal clinical course, diagnostic evaluation, and outcome are summarized in Table 1.

**Table 1:** Clinical timeline of peanut allergy history, evaluation, and delabeling SPT: skin prick test, IgE: immunoglobulin E, OFC: oral food challenge

Category	Childhood (~10-11 years)	Adolescence (~13+ years)	Early adulthood (19 years)
Presentation	Immediate lip swelling and dyspnea after peanut ingestion	Resumed peanut ingestion without symptoms	Referred for reevaluation for military accession
Diagnosis	Peanut allergy diagnosed (SPT performed; prior results unavailable)	No recurrent symptoms	Positive SPT (6 mm wheal); low-level peanut-specific IgE (2.16 kUA/L); isolated Ara h 8 sensitization (2.54 kUA/L)
Management	Prescribed epinephrine autoinjector (not used)	-	Supervised OFC tolerated
Outcome	-	-	Allergy delabeled; cleared for military accession

Skin prick testing during the evaluation demonstrated sensitization to peanut extract, with the largest wheal and flare diameters measuring 6 mm and 25 mm, respectively, and appropriate positive and negative controls. A wheal at least 3 mm greater than the negative control was considered positive [6]. Given the remote history of a childhood reaction involving multiple systems and persistent sensitization on skin testing, OFC was deferred pending further risk stratification. Serum testing revealed a peanut-specific IgE level of 2.16 kUA/L. CRD demonstrated sensitization to Ara h 8 (2.54 kUA/L), with undetectable IgE to Ara h 1, 2, 3, 6, and 9. Peanut allergen components, designated “Ara h” and derived from *Arachis hypogaea*, represent specific protein fractions used in CRD. PR-10 proteins, such as Ara h 8, are associated with cross-reactivity and typically mild or absent clinical reactions. This sensitization pattern is consistent with pollen-food allergy syndrome (PFAS), in which cross-reactivity between peanut PR-10 proteins (Ara h 8) and homologous birch pollen allergens may result in sensitization without clinically significant systemic reactions [7]. This interpretation is supported by the patient’s history of seasonal allergic rhinitis, although specific pollen sensitization data were not available.

Following review of laboratory results and shared decision-making, the patient underwent a supervised open OFC to peanut butter. The challenge was conducted using graded doses per institutional protocol and completed without symptoms. The patient tolerated a standardized full serving of peanut butter (2 tablespoons) and remained under observation for two hours post-ingestion without evidence of an allergic reaction [8]. Based on the successful challenge, peanut allergy was delabeled. The patient was advised that dietary avoidance and epinephrine carriage were no longer necessary, and documentation confirming tolerance was provided for military accession purposes.

## Discussion

Food allergy diagnoses may persist long after clinical reactivity has resolved, resulting in continued labeling despite established tolerance. Although many individuals labeled with peanut allergy later demonstrate tolerance, extract-based testing alone may perpetuate diagnostic misclassification, as sensitization on skin prick testing frequently persists even in clinically tolerant individuals [4,9]. Positive serologic and skin prick test results indicate sensitization, reflecting the presence of allergen-specific IgE, but do not necessarily correlate with clinical reactivity in the absence of reproducible symptoms [4]. In this patient, continued asymptomatic peanut ingestion over several years contrasted with persistent extract-based sensitization, underscoring the limitations of conventional testing when interpreted without integration of clinical history and adjunctive diagnostics.

CRD refines the evaluation of peanut sensitization by distinguishing IgE reactivity to storage proteins, such as Ara h 2, which are associated with systemic reactions, from cross-reactive proteins, such as Ara h 8, which are more commonly associated with milder or absent symptoms [5]. PR-10 proteins such as Ara h 8 are heat-labile and associated with cross-reactivity to environmental allergens (e.g., homologous to birch pollen Bet v 1), and often present as mild oropharyngeal symptoms consistent with pollen-food allergy syndrome, whereas seed storage proteins (Ara h 1, 2, 3, and 6) are heat-stable and more strongly associated with clinically significant systemic reactions [4]. Profilins such as Ara h 5 (Bet v 2 homolog) may also contribute to pollen-food cross-reactivity but are considered minor allergens with limited clinical significance [7].

In clinical practice, these distinctions support risk stratification and guide decisions regarding the appropriateness of OFC, which remains the gold standard for diagnosis [8,10]. Meta-analytic data demonstrate that CRD improves diagnostic accuracy compared with extract-based testing; however, results do not eliminate the need for clinical correlation and confirmatory OFC [11]. In this case, the patient’s ongoing tolerance of peanut without symptoms, combined with isolated Ara h 8 sensitization and absence of storage protein sensitization, supported progression to supervised OFC.

While no universally standardized approach to food allergy delabeling exists, a combined strategy incorporating longitudinal clinical history, CRD, and supervised OFC provides a practical framework for safely reassessing and removing inaccurate allergy labels.

In the United States, this issue has particular relevance for military applicants, where historical diagnoses of food allergy may lead to medical disqualification despite the absence of ongoing clinical reactivity [12]. Diagnostic misclassification in this setting may carry disproportionate professional consequences, as inaccurate allergy labeling can affect eligibility for service. Some applicants may report remote childhood reactions without subsequent symptoms yet continue to carry an allergy label [13]. Careful reassessment of such legacy diagnoses, incorporating contemporary diagnostic tools and confirmatory testing, may help distinguish true allergy from resolved or clinically insignificant sensitization in this population.

The evolving role of CRD in peanut allergy evaluation is particularly evident in cases where conventional testing and clinical history are discordant [14]. In this patient, CRD did not replace clinical judgment but refined it, helping contextualize persistent sensitization in the setting of ongoing tolerance and supporting progression to supervised OFC. As the use of CRD continues to expand, its integration into clinical decision-making remains complex, reinforcing the importance of combining laboratory data with longitudinal history and confirmatory testing when considering food allergy delabeling.

## Conclusions

Legacy food allergy labels, particularly those based on remote childhood reactions, warrant reassessment when clinical history suggests tolerance. In this case, supervised OFC confirmed tolerance and allowed for safe removal of an inaccurate peanut allergy label. These findings have important implications in settings where diagnostic misclassification carries meaningful consequences. Integrating clinical history with appropriate diagnostic testing is critical when evaluating presumed food allergy. Precision in food allergy diagnosis is not optional but essential.
